# Supplementary material for: Are carbon emissions trading and green financial instruments synergistic? -Comprehensive quantitative research based on content analysis
Source: PLoS One. 2024 Mar 7;19(3):e0298601. doi: 10.1371/journal.pone.0298601 (PMC10919601; doi:10.1371/journal.pone.0298601)
Supplement: S1 Data — (ZIP) [file pone.0298601.s001.zip › Data/地级市Z_F工作报告.zip!/║╙▒▒/╠╞╔╜╩╨/└┤╫╘╣1⁄2╓┌║┼í╛┬φ┐╦╩2╛▌═°í┐_2010.docx]

一、2009年工作回顾

刚刚过去的一年，是唐山发展史上很不寻常的一年。面对国际金融危机带来的严峻挑战，全市人民在市委的坚强领导下，坚决贯彻中央、省一系列决策部署，以科学发展示范区建设为总揽，全力实施“五项攻坚行动”和“八大工程”，统筹推进保增长、保民生、保稳定各项工作，坚定信心，迎难而上，扎实苦干，较好地完成了市十三届人大二次会议确定的各项目标任务，科学发展示范区和人民群众幸福之都建设向纵深推进。

(一)全力应对国际金融危机挑战，扩内需、保增长、调结构取得新突破。预计全市完成地区生产总值3800亿元，同比增长11%以上;全部财政收入413.3亿元，剔除增值税转型政策影响，按上年可比口径增长14.1%，其中一般预算收入169.7亿元，同比增长15.8%。坚持把保增长与调结构、促转型紧密结合起来，实施千个项目保增长调结构攻坚行动，取得明显成效。全社会固定资产投资完成2180.9亿元，增长60.1%;城镇新开工项目达到2240个、增长1.4倍，完工项目1397个。信贷规模大幅增长。全市金融机构各类存款余额3677亿元、贷款余额2211亿元，新增贷款653.8亿元，比上年增长1.1倍，增量存贷比达到86.3%，同比提高37.7个百分点。在宏观经济环境比较困难的情况下，规模以上工业增加值增长13.6%。产业结构调整步伐加快。钢铁产业整合改造取得重要进展，渤海、长城两大钢铁集团重组扎实推进;装备制造业规模化、集约化水平不断提高，时速350公里动车组、中低速磁悬浮列车、电动汽车等一批高新技术产品逐步实现产业化，我市和曹妃甸新区被国家列为信息化与工业化融合试验区;服务业增幅13%，占全市GDP比重达到34.6%，比上年提高3.5个百分点，全社会消费品零售总额增长18.4%。节能减排成效显著。全市淘汰落后炼钢产能360万吨、炼铁549万吨、水泥630万吨、造纸29.8万吨、小火电机组62.4万千瓦。预计万元生产总值能耗同比下降5.28%，化学需氧量和二氧化硫排放量分别削减1.8万吨和6.5万吨，三项指标均完成或超额完成省达目标。

(二)强力推进唐山湾“四点一带”开发建设，沿海经济隆起带支撑带动作用日益凸显。“四点一带”地区完成生产总值1162亿元，全社会固定资产投资1387亿元，分别占全市的31%和64%。其中,曹妃甸新区完成投资1023亿元。产业聚集步伐加快，首钢京唐钢铁厂一期投产，华润曹妃甸电厂2×30万千瓦机组并网发电，华电临港重型装备制造、冀东哈电风力发电装备制造、中恒科技太阳能电池、中兵光电产业基地、中视中科激光显示基地、第四方物流南堡现代物流园等46个项目开工建设，曹妃甸纯电动汽车、中石化千万吨级炼油等一批重大产业项目前期工作取得积极进展。曹妃甸港区煤炭码头一期、通用码头一期和二期工程投入运营，京唐港区3000万吨专业煤炭泊位竣工。唐山港货物吞吐量达到1.76亿吨、增长61.8%，在全国港口排名由2008年的第15位提升到第12位。曹妃甸中期区域用海总体规划已获批准，填海造地形成陆域面积200平方公里，建成标准厂房90万平方米，司曹铁路全线通车，滦曹公路、滨海大道开工建设，张曹铁路等工程前期工作进展顺利，路、水、电、讯等配套设施日臻完善。以曹妃甸为龙头的唐山湾地区成为新兴产业聚集的高地和新型工业化的重要平台。

(三)深入推进城镇面貌三年大变样，城市框架迅速拉开、功能不断完善、形象显著提升。全市城镇建设投资达到510亿元，是历年来投入最多、城市面貌变化最大的一年。 “四城一河”建设取得突破。南湖生态城开发建设加速推进，扩湖工程形成11.5平方公里水面，栽植全冠大树7.6万株、造林4100亩、种植草坪和灌木3100亩，地震遗址公园、市民广场等工程完工，紫天鹅庄酒店基本建成，南湖城市中央生态公园被联合国人居署授予“HBA中国范例卓越贡献最佳奖”、被中国生态文化协会授予首批“全国生态文化示范基地”称号;曹妃甸生态城完成起步区造地7平方公里，假日酒店、信息大厦等一批项目开工，规划展厅建成，央企服务基地一期具备入住条件;凤凰新城市政基础设施进一步完善，开发建设全面展开，交通路网基本形成，新唐山一中、新青少年宫、香格里拉大酒店等一批项目加紧建设;空港城起步区村庄搬迁改造、市政基础设施建设逐步展开，军民两用机场航站区主体工程完工;陡河青龙河改造全面启动，环城水系供水管线工程全面完工。旧城改造力度加大，拆违拆迁556万平方米;震后危旧平房改造新开工安置住房251万平方米，累计达到570万平方米，竣工200万平方米;实施城中村改造项目19个;既有居住建筑节能改造完成510万平方米。打通了光明路、大里路等13条断头路，完成了机场连接线、唐丰路入市通道建设和景观改造。“绿、美、亮、净”工程深入实施，城市形象和品位显著提升。组织开展了声势浩大的绿化攻坚行动，新增造林绿化面积60.5万亩，全市森林覆盖率提高2.25个百分点，顺利通过全国绿化模范城市验收。完成了全部县城和19个中心镇规划修编工作，县城扩容和小城镇建设加快推进。预计全市城镇化率达到53%，同比提高2个百分点，城镇化发展指数位居全省首位。

(四)坚持统筹城乡发展，新农村建设迈出新步伐。认真落实国家各项强农惠农政策，发放粮食直补、大型农机具购置以及家电、汽车、摩托车下乡等补贴资金7亿多元，相当于全市每个农户受益700元。粮食生产再获丰收，总产达304万吨，增长5.6%，连续6年实现增产。农业结构调整扎实推进，现代农业发展步伐加快。实施农业产业化带动项目120个，农业产业化经营率达到63%，同比提高2个百分点，畜牧水产业产值占农业总产值比重达到44.3%。农村基础设施建设进一步加强，新建改造农村道路1249公里、2000个村实现硬化道路户户通，在271个村实施农村饮水安全完善改造工程、惠及21万人。农村改革不断深化，成立市县农村土地经营权流转交易中心，构建了覆盖县、乡、村的土地经营权流转交易服务管理网络;在市县两级设立了农民进城受理服务中心。以推行新民居建设“六个一”模式为重点，科学发展示范村创建工作扎实开展。新民居建设开工175个村，累计完工645.7万平方米，旧民居改造开工200个村，农村人居环境有了新的提升，农村面貌发生较大变化。

(五)坚定不移地促开放抓改革，发展活力不断增强。成功举办了首届曹妃甸论坛，对外开放层次和水平实现新提升。论坛正式参会代表超过1000人，中共中央政治局常委、全国政协主席贾庆林亲自出席论坛并发表主旨演讲。这次论坛规模之大、规格之高、影响之广，在我市涉外活动历史上是少有的，提高了唐山的知名度和美誉度，成为推动唐山进一步扩大开放、全面走向世界的新起点。还先后举办了优秀民营企业家走进唐山、央企走进曹妃甸、百名侨领走进曹妃甸、海峡两岸企业发展与合作论坛、第二届曹妃甸临港产业国际合作会议、第十二届唐山中国陶瓷博览会等一系列招商推介活动，活跃了对外开放工作全局。在金融危机蔓延、国际需求萎缩的不利影响下，全市实际利用外资8亿美元，完成进出口总额61亿美元，均居全省首位。对内交流与合作取得新成效，先后与上海、四川锦阳和我省的承德、秦皇岛等市，中科院、清华大学、对外经贸大学等科研院所，以及中国华能、民生银行等企业集团签订了一批战略合作协议。国有企业和国有资产管理体制等项改革扎实推进。组建了冀东发展、唐山港口实业等国有企业集团，冶金矿山机械、盾石机械等企业搬迁改造启动实施。整合组建了市属投融资公司，投融资平台功能得到充分发挥，有力地促进了重大基础设施与城市建设改造、产业结构调整和民生改善。市政府机构改革基本完成，科技、文化、教育等改革取得实效。深入开展全民创业，民营经济快速发展，增加值占全市生产总值比重达到64.8%。

(六)统筹推进经济社会协调发展，各项社会事业全面进步。全市财政投入社会事业资金120亿元，增长14.4%。科技创新能力进一步增强，被确定为首批国家创新型试点城市。330项科技成果达到国内领先水平，中科院5个研究所进驻中科院唐山高新技术研究与转化中心，国家高速动车组高新技术产业化基地落户我市。教育事业全面发展。开放式素质教育加快推行，义务教育均衡发展，被评为全国首批义务教育均衡发展先进地区;唐山工业职业技术学院曹妃甸新校区开工建设，河北理工大学、华北煤炭医学院合并建设综合性大学并获博士学位授予权建设资格，与西南交大、复旦大学上海视觉艺术学院等高校的战略合作取得积极进展。 “健康唐山、幸福人民”行动全面展开。为全市91.3万人建立健康档案、为91万人制定健康计划、为92.4万人进行了免费体检;改扩建乡镇卫生院30所，村卫生室标准化率达到74%，城市社区卫生服务人口覆盖率100%;全力组织甲型H1N1流感疫情监测、预防和患者救治，深入开展城乡爱国卫生运动，保障了人民群众的健康安全。文化体制改革不断深化，文化事业蓬勃发展。整合市五大专业剧团，组建成立了唐山演艺集团公司，唐山博物馆改扩建、开滦国家矿山公园、河北省文化创意产业园等文化项目顺利实施。全民健身运动广泛开展，竞技体育水平进一步提高。我市健儿在第十一届全运会上获得五枚奖牌，奖牌总数超过上届。创建全国文明城市活动扎实推进，城市公共文明指数在国家测评中进入地级市前10名。人口自然增长率4.46‰，独生子女父母退休一次性奖励3000元政策在全省率先实施，被确定为全国首批人口和计划生育综合改革示范市。民族宗教、广播电视、外事侨务、防震减灾、妇女儿童、老龄、气象、档案、人防、民兵预备役和国防动员等各项事业全面发展。

(七)高度重视改善民生，人民群众幸福指数进一步提升。城镇居民人均可支配收入18053元，增长10.2%;农民人均纯收入7420元，增长12%。投资60亿元，为群众办的20件实事全部完成。全市城镇新增就业6.8万人，下岗失业人员再就业3.4万人，城镇登记失业率4.1%，实现了“零就业”家庭动态归零。将高校在校生、城中村人员、外来务工人员和家属子女纳入城镇居民基本医疗保险参保范围，城镇居民医保参保率达到96%，新型农村合作医疗保险参合率96.1%，在全省率先实现了“农民进城医疗报销无障碍”。城镇低保标准提高到每人每月285元、农村低保标准提高到每人每年1300元，新型农村养老保险试点扩大到8个县(市)区，企业退休人员养老金月人均提高到1144元。新开工经济适用住房67.8万平方米、开工(筹集)廉租房30.6万平方米。社会矛盾综合调控效果明显，大量信访问题及时妥善化解。始终保持对违法犯罪的高压态势，人民群众安全感进一步增强。食品药品安全整治扎实开展，食品药品安全水平不断提高。强力推进安全生产攻坚整治，采取党政“一岗双责”齐抓共管、高规格配备安委会、严格落实企业安全生产主体责任、持续开展安全生产专项治理、组织全员模拟培训、实施科技兴安、安全生产全民参与等七大举措，全市安全生产形势保持了总体稳定、持续好转的态势。

(八)深入开展“干部作风建设年”活动，政府行政效能和服务水平不断提高。按照省委、省政府和市委统一部署，认真开展“干部作风建设年”活动，努力在深入上下功夫、在实效上求突破。制定实施了市政府领导班子关于改进工作作风、狠抓工作落实的十三项措施，领导干部公开个人电子信箱、公开承诺重点工作等制度得到有效落实。认真开展政府规章、规范性文件和行政许可、非许可类行政审批事项清理规范工作，削减力度和承诺办理时限在全国同类城市中处于领先水平。全面落实网上审批、并联审批、超时默许和重点项目后备干部代办制，在全市政府机关大力弘扬“白加黑、五加二、重点项目建设三班倒”的工作精神，全面推行“一线工作法”，努力打响唐山效率品牌。建设和开通了城乡一体化服务管理信息系统，将全市原有22条政务公开服务热线整合为“唐山市人民公共服务热线”，搭建了服务群众的新平台。严格行政执法，推进行政权力公开透明运行，惩治和预防腐败体系建设取得新成效。自觉接受人民代表大会及其常委会的依法监督，重视发挥人民政协的政治协商、民主监督和参政议政作用，承办人大代表建议和政协委员提案609件，按时办结率达到100%，代表、委员满意率97%，有力促进了政府工作水平的提高。

各位代表，过去的一年是我市经济社会发展经受严峻考验的一年，也是科学发展示范区建设继续向前推进、取得令人鼓舞新成就的一年，成绩来之不易。这是党中央国务院、省委省政府和市委正确领导的结果，是全市人民众志成城、顽强拼搏、共克时艰的结果，是科学发展示范区建设不断深化、不断积累、不断跨越的结果。在此，我代表市政府，向全市工人、农民、知识分子、各级干部和广大建设者，向人大代表、政协委员，向各民主党派、工商联、无党派人士和人民团体，向驻唐部队、武警官兵、公安政法干警以及关心支持唐山发展的各界朋友，表示崇高的敬意和衷心的感谢!

总结过去的一年，我们也清醒地认识到，我市经济社会发展中仍存在着诸多亟待解决的问题。主要是：在当前宏观经济环境下，部分行业和企业生产经营仍比较困难，外贸出口下滑，财政收支矛盾突出，保持经济持续平稳较快增长的任务十分艰巨;经济结构性矛盾仍然比较突出，转变发展方式的任务繁重，特别是节能减排、安全生产压力较大，资源型城市转型需要付出更加艰苦的努力;保持农民收入较快增长的难度加大，新农村建设需进一步加快、水平需进一步巩固提升;发展成果的普惠性有待提高，劳动就业、社会保障、人居环境、公共安全等关系群众切身利益的问题还需下大力更好地解决;政府自身建设有待进一步加强，一些政府工作人员的工作理念、方式、作风还不能适应新形势的要求和人民群众的期望，服务意识不强、不作为、乱作为现象还不同程度存在。对这些问题，我们必须以对党和人民高度负责的态度，采取更加有力的措施，认真加以解决。

二、2010年工作思路和主要目标

今年是实施“十一五”规划的最后一年，是巩固经济回升向好势头、保持经济平稳较快发展、为“十二五”发展奠定良好基础的关键一年。做好今年工作，对于夺取应对国际金融危机冲击全面胜利、加快科学发展示范区和人民群众幸福之都建设具有重要意义。

展望2010年，机遇与挑战并存。从机遇看，纵观国际国内形势，经济社会发展环境将好于去年。国际金融市场渐趋稳定，世界经济有望恢复性增长;我国经济回升向好的基础逐步稳固，中央继续实施积极的财政政策和适度宽松的货币政策，保持宏观经济政策的连续性、稳定性，扩大内需和改善民生的政策效应将进一步显现。就我市来讲，当前正处在一个跨越发展的新时期。经过三年的科学发展示范区建设实践，全市上下思想观念、发展方式、城市形态、城乡结构、管理方式、人民群众生活品质发生了显著变化，呈现出政通人和、心齐气顺、风正劲足、经济发展、社会和谐的生动局面。特别是唐山湾“四点一带”、城市“四城一河”开发建设和一批重大项目的启动实施，为唐山长远发展积蓄了强大后劲，为我们在新的起点上加快科学发展示范区建设奠定了坚实基础。从挑战看，外部经济环境不确定、不稳定因素仍很多，世界经济复苏将经历缓慢复杂曲折的过程，国内经济增长的内在动力仍然不足。尤其是我们还面临着周边地区竞相发展带来的巨大压力，如果不奋起直追、奋力超越，就会在环渤海地区崛起中错失乘势而上、借力腾飞的大好机遇。对当前形势，我们一定要准确把握、积极应对，既要看到有利条件，抓住难得机遇，发挥综合优势，力促经济社会又好又快发展，又要保持清醒头脑，增强忧患意识，研究制定有效应对措施，增强工作的系统性、预见性和主动性，努力推动各项事业实现新的跨越。

年初召开的市委八届六次全会，对当前形势进行了科学分析，围绕贯彻党的十七届四中全会、省委七届五次全会和中央、省经济工作会议精神，基于对科学发展示范区建设的探索实践，对今年工作做出了战略部署，为推动全市经济社会又好又快发展、加快科学发展示范区建设进一步指明了方向。我们一定要按照市委统一部署，坚定信心、振奋精神，拼搏进取、埋头苦干，努力把唐山改革发展推向一个新水平。根据市委八届六次全会精神，今年全市国民经济和社会发展的总体要求是：认真贯彻落实党的十七大和十七届四中全会、省委七届五次全会精神，深入贯彻落实科学发展观，以科学发展示范区建设为总揽，把保持经济平稳较快发展与加快经济发展方式转变有机统一起来，把握“创新与项目建设年”这一工作主题，推动发展观念、发展速度、发展方式“三个跨越”，实施科教立市、生态立市、港口立市、制度立市“四项战略”，统筹推进新型工业化、新型城镇化、城乡等值化、社会治理和谐化，打好以曹妃甸为龙头的唐山湾“四点一带”产业聚集、以“四城一河”为重点的城镇面貌三年大变样、结构优化升级、县域经济发展、城乡统筹发展、改善民生“六项攻坚战”，全面完成“十一五”各项目标任务，把科学发展示范区和人民群众幸福之都建设推向一个新水平。

全市经济社会发展主要预期目标是：地区生产总值增长10%;全部财政收入增长10%，其中一般预算收入增长10%;城镇居民人均可支配收入增长8%，农民人均纯收入增长10%;全社会固定资产投资增长30%;万元生产总值能耗降低3.42%以上;二氧化硫排放量减少3%、化学需氧量排放量减少3%;城镇登记失业率控制在4.5%以内;人口自然增长率控制在5.6‰以内。

上述发展目标，是在全面分析国际国内形势、科学把握市情市力的基础上，衔接“十一五”规划目标和科学发展示范区建设任务，经过科学测算确定的。只要全市上下统一思想、团结一心、艰苦奋斗，是完全可以实现的。

三、2010年主要工作任务

市委八届六次全会明确提出，把科学发展示范区建设推向深入，在工作中就是要在做强新型工业化、新型城镇化、城乡等值化、社会治理和谐化和党的建设科学化“五大支柱”上下功夫，不断取得新的突破。我们必须全面贯彻落实市委战略部署，立足当前、着眼长远，强化措施、全力推进。

(一)围绕转变发展方式，着力推进新型工业化。工业化是现代化的必由之路。坚持绿色增长的发展方向，以项目建设为载体，以结构调整为主线，以沿海临港产业发展为龙头，以节能减排为抓手，以科技创新为动力，实施产业结构调整三年攻坚，加速形成以高新技术为支撑的精品钢铁、装备制造、化工和新能源、环保、生物医药两个“三足鼎立”的产业发展新格局，打造具有唐山特色的新型工业体系，努力走出一条资源节约、环境友好、创新驱动、人力资源优势得到充分发挥的新型工业化道路。

狠抓重点项目建设。继续实施千个项目保增长调结构攻坚行动，全市安排重点项目1058项，总投资1.07万亿元，年度计划投资2010亿元。突出抓好“四个一批”：开工一批，力促曹妃甸电动汽车、丰润装备制造园区石油套管等400个项目开工建设。完工一批，确保华电临港重型装备制造、承唐高速二期等200个项目完工或部分完工。推进一批，加快中石化曹妃甸千万吨级炼油、京唐城际高速铁路等100个重点项目前期工作，确保取得实质性进展，尽早启动建设。继续做好中央新增投资项目的申报争取和建设工作。谋划一批，遵循国家产业政策，围绕产业优化升级、基础设施建设、城市建设改造、改善民生等重点领域，抓紧谋划和储备一批大项目、新项目，严格控制高耗能、高污染和产能过剩项目。充分发挥企业主体作用，引导启动好民间投资，保护投资者合法权益。优化项目投资环境，保障重点项目用地、资金等要素需求。用足用好国家信贷政策，抓住国家继续实施适度宽松货币政策的机遇，围绕信贷投放重点，积极创造条件，力争全年新增贷款500亿元以上。加快唐山商业银行战略重组，力争华夏银行、浦发银行等更多的外埠银行进驻唐山;鼓励企业上市和发行债券，探索设立股权投资基金，积极建立创业投资(基金)公司，规范发展小额贷款公司等金融组织，提高金融对经济发展的支撑能力。

大力推进传统产业优化升级。抓住我市被列为国家信息化与工业化融合试验区的机遇，瞄准中外先进技术和水平，开展对标行动，加大用先进适用技术改造传统优势产业力度，做优做强三大支柱产业。钢铁产业，按照“控制总量、淘汰落后、企业重组、技术改造、优化布局”的思路，以首钢、唐钢、渤海、长城、津西等大型骨干企业集团为龙头，加快整合重组步伐，引导产业向南部沿海地区转移、向各类园区集聚，尽快构建起产业集群化、装备现代化、产品精品化、资源利用循环化的现代钢铁产业体系，加快向钢铁强市迈进。装备制造业，以建设国家重型装备制造基地和现代制造业配套基地为目标，以丰润中国动车城、开平现代装备制造园和曹妃甸装备制造园等产业园区为依托，加快实施高速列车生产配套装备、重型装备等一批重大装备制造项目，做大做强产业集群。化工产业，以推进曹妃甸大型石油炼化项目为突破口，积极发展高附加值、精深加工特色产品链，加快构建石化、煤化、盐化“三化合一”产业新格局。推动水泥、陶瓷等传统产业技术改造升级，增强市场竞争力。

着力培育发展新兴战略产业。着眼于构建唐山未来发展的战略支撑，以新能源、环保、生物医药产业为重点，加快发展新兴战略产业，形成绿色增长新板块。新能源产业，大力发展电动汽车、风力发电设备制造、光伏发电、半导体照明等产业，构筑国内一流新能源产业集群。环保产业，积极引进瑞典、日本等发达国家先进技术，重点开发大气、水和固体废弃物污染防治，清洁生产、循环经济和资源再利用技术与工艺设备等产业。生物医药产业，利用高新技术，加快培育发展以生物制剂、化学原料药深加工、现代中药、生物制造和生物能源为主体的生物医药产业。同时，积极培育电子信息和文化创意产业。

高度重视现代服务业发展。全面落实鼓励支持政策，在提升商贸流通等传统服务业的同时，大力培育壮大现代物流、金融服务、会展等生产性服务业，积极发展旅游、文化、商务服务等新兴服务业。坚持把现代物流业作为服务业发展的重中之重，抓住我市被列为全国区域性物流节点城市的机遇，加快曹妃甸、京唐港、空港城三大物流园区和装备制造、钢铁两大生产服务型物流集聚区建设，推动现代物流业上规模上水平。全市物流业增加值增长40%以上。加快发展旅游业，进一步完善规划、整合资源、塑造品牌，大力发展红色游、工业游、乡村游、海滨海岛游、皇家文化游和体育休闲游、生态湿地游，突出抓好南湖城市中央生态公园、唐山湾“三岛”开发等一批重点旅游项目建设，努力把唐山打造成环京津休闲旅游活力地区，加快建设国家知名旅游城市。

加快沿海临港产业聚集发展。围绕壮大新型工业化龙头，以曹妃甸为重点，以产业聚集为核心，掀起唐山湾“四点一带”开发建设新高潮。今年唐山湾“四点一带”地区全社会固定资产投资达到2000亿元，其中曹妃甸新区完成1500亿元，分别增长45%和50%。坚持把产业聚集放在突出位置，全面加快曹妃甸县(市)区临港产业园区和各专业园区建设，加大招商引资力度，力促项目落地开工，打造沿海产业集群带。围绕建设绿色港口目标，完善落实绿色港口建设指标体系，抓好矿石码头二期、通用码头三期、煤码头续建等工程建设，力争矿石码头三期、煤码头三期开工建设，唐山港货物吞吐能力突破2亿吨。完善路网、供水、供电等配套基础设施，确保滨海大道全线贯通，谋划启动南曹快速路、滨海大道西延等项目建设，提高产业承接能力。

强力推进节能减排。今年是全面落实“十一五”节能减排目标的最后一年。以更加坚决的态度、更加过硬的措施、更加有力的手段，继续深入实施省“双三十”和市“10100”工程，确保完成既定目标任务。以曹妃甸国家级循环经济示范区、司家营循环经济示范区为龙头，以钢铁、化工、建材等行业为重点，推行绿色低碳发展模式，大力发展循环经济。加强节能减排项目建设，狠抓完工项目正常运营，新谋划实施100项节能项目、100项减排项目。全面落实目标责任制，加强节能减排监测监察，严格“两高”项目禁(限)批管理，广泛推行清洁生产，推进新装备、新工艺、新技术的应用。严格执行国家产业政策，坚决淘汰落后生产能力，最大限度地提高资源能源利用率、减少环境污染。

加速科技创新和技术进步。坚持政府推动、企业主体、市场导向，政产学研金介相结合，广聚创新资源，不断提高自主创新能力，加快构建完善的科技创新体系和开放式的科技创新机制，全面推进国家创新型试点城市建设。全市财政安排科技资金3.6亿元，重点实施4个重大科技专项，转化150项重大专利技术，加快智能机器人等一批具有自主知识产权的高新技术产品产业化步伐。加强电动汽车、循环经济技术、生态城市等工程技术研究中心建设，打造科技创新研发平台;积极推进唐山科技城建设，启动建设唐山环渤海知识市场，打造科技创新服务平台;推进中科院唐山高新技术研究与转化中心建设，引进中科院研究所达到8至10家，打造科技创新合作平台。通过打造“三个平台”，促进先进技术成果向现实生产力转化，推动产业优化升级和新兴战略产业发展壮大。积极争取列为国家“十城千辆”和“十城万盏”试点城市。

(二)围绕建设生态城市，着力推进新型城镇化。城镇化是实现经济社会持续快速发展、加速现代化进程的重要动力。以建设生态城市为目标，以提高城镇综合承载能力为重点，以城市建设改造为抓手，全方位提高城镇化发展水平。

落实生态理念。以曹妃甸生态城141项生态城市指标体系为样板，通过制度规范、政策引导、政府推动，把指标体系贯彻到城镇规划、建设、管理的各个环节。加快城市“四大功能区”等新城建设，努力打造生态新城区。积极利用节能环保新材料、新技术，推进市县旧城区生态化改造。以南湖为模板，加强对采煤沉降区、工业废弃地的生态治理和修复，打造生态城市新板块。持续开展绿化唐山攻坚行动，新增造林绿化面积56万亩，森林覆盖率达到30%。全力做好2013年中国花卉博览会和2016年世界园艺博览会申办和筹备工作。

完善城镇布局。围绕构建以大中城市为主导、大中小城市和小城镇协调发展的城镇化体系，落实“双核两带”空间布局和总体思路，同步推进“大城市”、“大县城”和“中心镇”建设。加快完善主城区功能，稳步扩大规模，逐步发展成为300万人口的大城市;加快曹妃甸生态城建设，努力建成生态示范城市;加速县城扩容升级、扩大人口规模，努力朝着中等城市目标迈进;加快中心镇建设步伐，每个县(市)区培育1至2个5万人口以上中心镇，充分发挥辐射带动作用。

提升城市功能。按照建设宜居宜业的现代化城市目标，全面提速“四城一河”开发建设，加大旧城改造力度，加强市政公用设施建设，使城市发展功能、服务功能、人居功能、休闲功能更加完善。南湖生态城，以中央生态公园全面升级、西部和北部片区全面开发、东部片区全面启动为重点，加快推进基础设施、体育休闲、旅游服务、房地产开发、软件与文化创意等项目建设，使南湖开发建设再上新台阶。曹妃甸生态城，以市政工程、公共设施建设为重点，全面完成起步区路网建设，启动轨道交通、生态城医院等一批城市功能项目，加快科教城建设，尽快形成起步区城市基础框架。凤凰新城，进一步完善基础设施建设，加快新青少年宫、总部基地等重点项目建设进度，争取中央带状公园、金融街、大型城市商业综合体等一批项目开工建设。空港城，尽快实现军民两用机场正式通航，加快路网等市政基础设施建设和起步区村庄搬迁改造，力争一批产业项目尽快落地。陡河青龙河改造，57公里环城水系全线贯通，完成全线景观改造和绿化建设，打造滨水生态景观带和服务业产业带。推进大城山区域开发，确保取得实质性进展。抓好城市路网、新火车站区域开发、百货大楼周边区域改造、城中村改造等旧城改造重点工程。进一步加强旧城区的保护和修缮工作。

强化城市管理。以权力下放、责任到人、监督到位和采取现代化手段为重点，进一步理顺管理体制，创新管理手段，提升管理水平。以政府机构改革为契机，将管理重心下放到区、街道，实现责权利相统一。完善市、区、街、居四级一体化公共服务管理，建立城市综合管理体系，强化检查和考评，提高城市精细化、网格化、数字化管理水平。加强社区物业管理，健全社区基层组织建设。持续开展“绿、美、亮、净”城市环境综合整治，不断提升城市形象。

科学经营城市。树立科学经营城市理念，推进城市资源资本化、资源配置市场化。健全政府一级开发土地机制，完善城市建设用地一级市场收储经营办法，依法依规管好、集约节约用好土地资源。规范房地产市场秩序，促进房地产业健康稳定发展。充分发挥各种融资平台作用，推行BT、BOT等先进经营运作模式，积极引进战略投资者，鼓励各类市场主体参与城镇开发建设，加快建立政府引导、金融机构支持、社会各方面参与的城市建设投融资体制。

(三)围绕实现城乡统筹发展，着力推进城乡等值化。城乡等值化是推动科学发展的内在要求。深入落实中央一号文件精神，坚持城乡统筹发展，以城乡等值化为目标，以工业化带动城乡统筹发展、以城镇化拉动城乡统筹发展、以产业化推动城乡统筹发展、以市场化促动城乡统筹发展、以信息化驱动城乡统筹发展。建立公共财政、公共服务、公共管理、公共政策等四项保障机制，加快城乡规划、建设、产业、公共服务和社会管理“五个一体化”。

加速推进现代农业发展。全面落实各项强农惠农政策，稳定粮食生产。实施设施农业拓展、规模养殖推进、生态农业推广、观光休闲农业示范、农民专业合作社规范、土地流转加速、龙头企业提升和食品加工业壮大、农业品牌创新、绿化唐山持续攻坚、农业生产条件改善等十大工程，加快农业发展方式转变，促进农业增效、农民增收。全市销售收入超亿元的大型龙头企业达到25家，农业产业化经营率达到64%。加快发展与城市现代工业相配套的生产加工业和服务业，促进城乡三次产业有机融合，拓展农民增收空间，努力增加农民非农产业收入。

着力改善农村人居环境。着眼于建设环境优美、设施配套、居住舒适的农民新家园，全面提升农村规划建设水平。通过集中联建、就地改建、易地新建等形式，建设一批居住相对集中、土地相对集约、各类要素相对集合的中心村。开展新民居建设和旧民居改造，完善道路、供水、排水等配套设施，改善农民生产生活条件。改造农村公路500公里、危桥1050延米，新增600个村实现硬化道路户户通。实施乡村环境整治工程，改善农村生态环境。

提升农村公共服务水平。加快城市公共服务向农村延伸，让广大农民群众共享改革发展成果。调整和优化城乡教育资源布局，促进优质教育资源共享。加快发展农村医疗卫生事业，开展“万名医师支援农村卫生工程”。积极推动农村医疗、养老等社会保障提标扩面，促进城乡社会保障制度接轨。加强农村文化设施和文化队伍建设，实施文化信息资源共享工程，推进乡镇综合文化站和农家书屋建设。进一步搞活农村金融，积极稳妥地发展村镇银行和小额贷款公司，建立健全信用担保体系，支持各类金融组织向农村延伸网点和机构，强化金融对农村发展的服务功能。

不断深化农村改革。稳定和完善农村基本经营体制，在依法自愿有偿的基础上，促进土地适度规模经营和土地承包经营权流转。积极探索农村股份制经营模式，通过资金、技术、劳动、土地等要素入股开展合作经营。继续推进征地制度改革，深化集体林权制度改革。支持农民创办经济实体和发展专业合作组织，壮大农村经纪人队伍，带动千家万户进入市场。鼓励村集体盘活现有资产、资源，发展集体经济，增加集体积累。

加快农村城镇化步伐。遵循城镇化发展规律，尊重农民意愿，完善城乡建设规划和镇村布局规划，全面完成乡村建设发展规划。制定《唐山市近郊城市化发展规划》，推动近郊率先实现城市化。加强政策支持，推进中心镇建设，努力建成小区域特色经济中心、城乡基本公共服务平台、城乡管理体制创新示范区。加强农村富余劳动力技能培训，深入实施农村劳动力转移培训“阳光工程”，提高农民就业创业能力。全年培训农民10万人，转移农村富余劳动力15万人次。深化户籍制度改革，全面落实农民进城落户政策，实现在城镇落户的农民与城镇居民同城同待遇，推动进城农民逐步融入城镇。

(四)围绕提高人民群众幸福指数，着力推进社会治理和谐化。社会和谐是科学发展示范区建设的重要目标。以改善民生为主题，以维护稳定为保障，以和谐文化建设为支点，以社会事业发展为重点，在推动和谐发展上不断取得新成效。

千方百计稳定和扩大就业。把就业摆在更加突出的位置，继续实施积极的就业政策，建立政府投资和重大项目建设与增加就业联动机制，多渠道增加就业岗位。积极开展就业援助，落实困难企业岗位补贴、社保补贴、培训补贴政策，加强就业指导和服务，重点解决好“零就业”家庭、低保对象和就业困难高校毕业生的就业问题。深入实施全民创业行动，抓好创业型城市创建工作，以创业带动就业。全年城镇新增就业岗位7万个以上，安置就业困难群体5000人以上，创业带动就业1.9万人，“零就业”家庭动态归零。

健全社会保障体系。实施社保惠民工程，推进社会保险扩面，将更多的中小企业就业人员、灵活就业人员、农民工纳入社会保险覆盖范围，基本实现城镇职工养老保险、医疗保险、工伤保险、失业保险制度全覆盖。继续提高企业退休人员养老金水平。开展城镇职工基本医疗保险和工伤保险市级统筹，确保企业养老保险省级统筹平稳过渡。开展职工重大疾病医疗互助活动。积极发展城乡社会救济和福利慈善事业，多渠道筹集和积累社会保障基金，构建弱势群体救助帮扶机制,切实保障困难群众基本生活。

优先发展教育事业。健全义务教育经费保障机制，加大教育投入力度，促进各类教育均衡发展。巩固和提高义务教育，推动开放式素质教育试点扩面。改善城乡中小学办学条件，全面实施中小学校舍安全工程。优化普通高中教育资源，新唐山一中建成投入使用。大力发展职业教育，实施规模倍增计划，用三年时间招生规模实现翻番。加快职业教育资源整合，唐山工业职业技术学院曹妃甸新校区力争年内主体工程完工。推动20所职业院校创建国家级、省级示范校和示范性实训基地。提升高等教育发展水平，加强强势学科和特色专业建设，进一步扩大对外交流合作，增强服务地方经济社会发展的能力。完善成人教育体系，加快发展民办教育。落实好教师绩效工资制，加强教师队伍建设，提高教师整体素质。完善家庭经济困难学生资助体系，确保家庭经济困难学生得到有效资助。

加快医疗卫生事业发展。按照国家、省统一部署，稳步推进医药卫生体制改革，力争在基本医疗保障制度、落实国家基本药物制度、健全基层医疗卫生服务体系、促进基本公共卫生服务逐步均等化、推进公立医院改革试点等方面取得实质性进展，为广大群众提供更加优质的医疗服务。加快卫生基础设施建设，启动建设市中医院病房楼、妇幼医院儿童病房楼、凤凰新城新工人医院，进一步提高医疗保障水平。搞好传染病预防控制，加强甲型H1N1流感、手足口病等重大疾病防控和救治工作。强化食品和药品安全监管，让广大群众买得放心、吃得安全。

加强和谐文化建设。继承和发扬唐山优秀文化传统，在全社会大力弘扬“感恩、博爱、开放、超越”的新唐山人文精神，打造城市之魂，使之成为建设新唐山的强大动力。加强社会公德、职业道德、家庭美德和个人品德教育，在全社会倡导“生命至尊、公平至上、诚信至要”的价值观念，以和谐文化引领和谐社会建设。坚持全国文明城、全国卫生城、全国健康城“三城联创”，广泛开展群众性精神文明创建活动，全面提高居民素质和城市文明水平。繁荣发展文化事业和文化产业，加快唐山博物馆改扩建等项目建设，启动建设市文化广场、传媒大厦、文化创意产业基地等文化基础设施和产业项目。弘扬冀东优秀地域文化，打造文化精品。办好第七届中国评剧艺术节。大力推动社会、企业、乡村、家庭文化建设，丰富和活跃群众文化生活。

积极发展体育、计生等各项社会事业。认真贯彻落实国家《全民健身条例》，广泛开展全民健身运动。以备战省第十三届运动会为契机，提高竞技体育水平。加快发展体育产业，启动奥体中心和南湖体育休闲基地建设。实施优生促进工程，提高人口素质，保持低生育水平长期稳定。继续抓好国防动员、人民防空工作，加强民兵预备役建设，深入开展“双拥”共建活动，积极探索推进军民融合式发展，继续创建全国“双拥”模范城。全面贯彻党的民族宗教政策，促进各民族共同团结奋斗、共同繁荣发展和宗教领域和谐稳定。协调发展广播电视、外事侨务、气象、地震、档案、老龄、妇女儿童、残疾人等各项事业，促进社会全面进步。

坚持不懈地抓好安全生产和食品药品安全。把安全生产作为“天字号”工程,强化领导，强化措施，强化责任，真正形成“党委领导、政府监管、行业管理、企业负责、社会监督”的安全生产工作格局。继续加大“七大举措”实施力度，尤其是严格落实安全生产责任制，强化企业主体责任的落实，对忽视安全生产的单位和企业，依法加大行政执法和经济处罚力度。按照大安全理念，把所有行业、企业、工地纳入监管视野，不间断开展安全生产专项整治行动，不间断加大安全生产投入，不间断组织教育培训，不间断进行执法检查，严厉打击各种安全生产非法行为，坚决防止重特大安全事故发生。加强食品药品安全长效机制建设，狠抓生产企业和流通行业整治，确保不发生重特大食品药品安全事故。

全力维护社会和谐稳定。高度重视公共安全，健全突发公共事件应急反应机制，加强环境污染、重大传染病防控等应急体系建设，提高预防和处置突发公共安全事件的能力。实施综合评估、综合预防、综合治理、综合化解、综合改革、综合考评，着力化解老矛盾，有效预防新矛盾。推行信访稳定工作综合调控新机制，深化体制、流程、方法、管理、问责“五个创新”，认真做好人民群众来信来访工作，切实解决好关系群众切身利益的突出问题。更新社会管理理念，突出社会管理重点，创新社会管理方式，健全完善社会管理体系。强化社会治安综合治理，依法严厉打击各种违法犯罪活动，进一步提高对社会治安秩序的控制能力，不断增强人民群众的安全感。今年建成主城区科技安防系统。

(五)围绕增强科学发展的动力和活力，着力深化改革、扩大开放。改革开放是经济社会发展的不竭动力。推进新型工业化、新型城镇化、城乡等值化和社会治理和谐化，加快建设科学发展示范区和人民群众幸福之都，必须坚定不移地深化改革、扩大开放。

加快推进重点领域改革创新。深化国有企业改革，加快打造一批拥有知名品牌和自主知识产权、主业突出、竞争力强的大型企业集团，抓好北方瓷都陶瓷、重型装备制造、交通运输等企业集团的组建整合。支持中小企业壮大发展，培育一批机制灵活、专业化分工明确、成长性强的中小企业。放手放开发展民营经济，全面落实鼓励民营经济发展的政策措施，支持民营企业增加投入、转型升级、做强做优。深化行政管理体制改革，全面完成市县两级政府机构改革，进一步减少和规范行政审批事项，提高行政效能。深化财政体制改革，大力培植税源，科学组织收入，强化预算管理，科学高效使用好财政资金，严格控制一般性支出，制定出台对县(市)区新一轮激励性财政体制政策。深化投融资体制改革，规范整合市县投融资平台，完善监管机制，强化政府债务管理。深化文化体制改革，推进经营性文化单位改企转制和公益性文化事业、经营性文化产业管理体制机制创新，整合组建广电传媒集团，支持唐山演艺集团公司壮大发展。继续深化科技、教育、社会管理等各项改革。

加快构建大开放新格局。坚持把扩大开放作为科学发展示范区建设的一项重大战略，强化开放意识，全面落实市委关于进一步扩大开放的若干重大措施意见，营造全民促开放的浓厚氛围，加快形成面向世界、面向全国、面向所有投资者的大开放格局。实施重点突破、全面开放，在积极推进与欧美国家合作的同时，以日、韩、新加坡和港、澳、台地区为重点，开展多领域、多层次的交流合作，下大力引进战略投资者，努力提升全市对外开放的规模、层次和水平。积极落实招商引资扶持奖励政策，改进招商方式，进一步扩大“招商月”活动成果，增强招商的针对性和实效性。全年实际利用外资8.5亿美元。办好第三届曹妃甸临港产业国际合作会议、第十三届唐山中国陶瓷博览会等重大经贸招商活动，确保收到实效。巩固扩大首届曹妃甸论坛成果。加强与环渤海经济圈、环京津有关省市的区域经济联合与协作。尤其是加强与京津沪在人才、资金、技术、产业、项目等领域的对接，不断扩大合作范围和领域，实现优势互补、互利共赢。积极扩大出口，加大对外贸企业的扶持力度，鼓励有市场竞争力的出口企业抢占国际市场，全市出口总额增长5%以上。支持有条件的企业“走出去”，更好地利用两个市场、两种资源，实现壮大发展。

各位代表，全面完成今年各项任务，推进新型工业化、新型城镇化、城乡等值化、社会治理和谐化，把科学发展示范区建设不断推向深入，必须突出重点，狠抓关键，强力攻坚。今年，集中力量打好六项攻坚战:

一是打好以曹妃甸为龙头的唐山湾“四点一带”产业聚集攻坚战。组织实施九大战役，力争曹妃甸新区完成投资1500亿元，增长50%以上。石油炼化攻坚战役，下大力推进中石化1000万吨炼油项目各项前期工作，争取列入国家“十二五”专项规划，力争早日开工建设;加快原油商业储备基地等项目建设。装备制造业园区攻坚战役，围绕水泥装备、新能源汽车、游艇制造等重点领域，加快一批龙头项目建设，力争锂源电动车、与上汽集团合作的绿色能源汽车、海天能源新城等10个项目开工建设，3至5个关联项目落地，尽快形成产业群体规模。电子工业园区攻坚战役，依托中科院中视中科激光显示核心产业基地，积极发展激光显示源模组等产业，力争一批电子信息产业项目年内竣工，一批新项目入驻园区。环保产业园区攻坚战役，瞄准节能、环保、资源循环利用等领域，大力发展环保装备、产品、技术、服务等产业。中日循环经济产业园区攻坚战役，按照建设节能、环保、循环经济产业聚集区的要求，加快推进与日本政府和企业间的合作，引进一批日资项目，开展一批技术合作。县(市)区临港产业园攻坚战役，基本完成园区造地和基础设施“七通一平”，力争每个园区至少有2至3个产业项目入园。曹妃甸生态城建设攻坚战役，抓好51个重点项目建设，启动30平方公里区域内路网建设，确保欧洲风情小镇、低碳建筑示范项目等一批项目取得突破性进展。大交通路网建设攻坚战役，确保滨海大道竣工通车，冀蒙铁路、南曹快速路等一批项目开工建设，形成大交通路网格局。配套推进各项基础设施建设。首钢二期工程攻坚战役，加速推进首钢二期各项前期工作，力争纳入国家“十二五”规划，2011年开工建设。

二是打好城镇面貌三年大变样攻坚战。今年是城镇面貌三年大变样工作的决战之年。围绕建设现代化生态城市、加速新型城镇化，以更大的魄力、更高的水平、更强的力度，把“三年大变样”工作进一步引向深入。全年城镇建设计划投资990亿元，增长近1倍。重点抓好南湖生态城、凤凰新城、曹妃甸生态城、空港城开发建设、陡河青龙河开发治理、唐山湾“三岛”开发建设、大城山周边区域开发、人居条件持续改善、中心区旧城改造、县城扩容升级等“十大工程”，确保取得新的突破。建设文化广场、市民中心、曹妃甸论坛会址、奥体中心、南湖老唐山风情小镇、中国近代工业博物馆、新火车站、曹妃甸可持续发展展示中心、大型城市主题雕塑、南湖之门等“十大标志性建筑”，总投资109亿元，年内完成主体工程，部分项目竣工投入使用。推进城市规划、城市功能、城市容貌、城市经营、城市管理“五大提升”，把城市规划建设管理提高到一个新水平。

三是打好结构优化升级攻坚战。强力实施“三百计划”，全面开展对标行动，确保在改造提升传统产业、高新技术产品研发和产业化方面取得实质性进展。实施“三百计划”。加快培育开发100项优质、高附加值、有市场需求的新产品，加速实现产业化;加快运用100项高新技术和先进适用技术改造提升传统产业，积极推进信息化与工业化融合发展，提升钢铁、水泥、化工、装备制造等传统产业技术装备水平和核心竞争力;加快培养引进100名支撑结构调整领军人才，健全和改进业绩评价、收入分配、服务保障机制，创优人才发展环境，确保领军人才留得住、用得上、出成效。开展以“创新超越”为主题的对标行动。在全市工业领域以企业对标为核心，以规模以上企业为主体，以钢铁、装备制造、化工、电力、水泥、陶瓷等行业企业为重点，选择省内、国内、国际同行业三个层面先进标杆，制定赶超规划和具体措施，对标赶超，跨越发展。力争通过三年左右的努力，创造国内100项一流指标、打造50家国内一流企业，全国县域经济百强县达到5家，全市工业经济在全国中心城市中进入前10强。

四是打好县域经济发展攻坚战。以项目建设为载体，以特色产业发展为依托，以园区建设为平台，全面提速县域经济发展。加大项目投资力度。各县(市)区全年全社会固定资产投资增幅达到30%以上;突出抓一批投资规模超亿元、十亿元的立县立区大项目、好项目。做强县域特色产业。引导各县(市)依托本地资源禀赋和产业优势，培育一批县域龙头企业、建设一批特色产业基地，提升产业发展集中度和竞争力，壮大县域经济规模，增强发展后劲。着力加强园区建设。按照“产业集聚、企业集群、特色鲜明”的目标，突出抓好各县(市)区工业园区特别是曹妃甸县(市)区临港产业园区建设，加大招商和产业集聚力度，每个园区确保有一批新产业项目入园。

五是打好城乡统筹发展攻坚战。以列为全省城乡一体化唯一一个试点市为契机，突出抓好“七个一批”，提高城乡统筹发展水平。抓一批城郊城镇化发展示范点。以主城区扩大带动近郊城市化，探索近郊区“村改居”、“农民变市民”的新机制，集中建设居民小区，全市重点培育5个示范点。抓一批县城扩容升级建设示范点。加快规划编制，拓展县(市)城规模，加速人口聚集，每个县(市)城年增人口5000人以上。抓一批中心镇建设示范点。在继续抓好18个中心镇建设试点的基础上，选择5个中心镇作为培育重点，进一步提升中心镇建设水平。抓一批大村庄建设示范点。全市重点培育10个大村庄建设示范点。抓一批土地经营流转示范点。规范农村土地承包经营权流转，促进土地适度规模经营。每个县(市)区抓1至2个具有一定规模、规范化的土地经营流转示范点。抓一批新民居建设。广泛推广新民居建设“六个一”模式，全面启动1000个村新民居建设。抓一批现代农业基地建设。每个县(市)区建成一个集生产、示范、观光为一体的现代农业园区，提升农业特色化、集约化、现代化水平。

六是打好改善民生攻坚战。围绕推进“十大幸福工程”，全年计划投入140亿元，重点为群众办好20件实事。在改善居住条件上，新开工市区危旧平房改造安置住房205万平方米，市中心区安置1万户以上;开工建设廉租房11万平方米，其中市中心区10万平方米;完成既有居住建筑节能改造500万平方米。在全民健康上，继续开展“健康唐山、幸福人民”行动，重点实施健康宣教、健康服务、健康饮食、健康文体、健康环境、健康生活、健康“细胞”、健康社会、健康信息“九大工程”，年内为全市45岁以上农民和城镇无业居民免费体检。在城乡养老和低保上，全面推进新型农村养老保险试点工作，扩大覆盖范围，让更多农民受益;提高城乡低保标准，全市城镇低保标准提高到每人每月310元，农村低保标准提高到每人每年1540元。在基本医疗保障上，稳步提高城乡医疗保障水平，将新农合筹资标准由每人每年100元提高到140元，其中财政补助标准提高到每人每年120元。在提升教育办学水平上，全市中小学多媒体普及率达到100%;新增远程教育项目学校180所，中小学远程教育覆盖率达到85%以上。在城乡环境保护上，深化钢铁、焦化、水泥等重点行业的污染治理达标建设，抓好25家企业烧结机脱硫工程，加大环境综合整治力度，全年城市空气质量二级及优于二级天数稳定保持在320天以上。在改善农村生产生活条件上，实施高标准农田建设示范工程，建设高标准农田11.2万亩;抓好农村环境道路硬化、院街净化、村庄绿化，新完成文明生态村建设500个;推行乡村垃圾清运处理，推广户收、村集、县(乡)集中处理模式，努力为农民群众创造清洁、健康、环保的生活环境。在便民服务上，在全市推广完善覆盖城乡的集社会保障、交通出行、公共事业缴费、小额支付消费、金融服务等功能为一体的“市民卡”，实现“一卡通”;新建社区市民中心78个，实现城市社区市民中心全覆盖;对全市10家城市标准化菜市场和10家农村农产品批发市场、农贸市场实施改造升级。在平安唐山建设上，启动建设社会治安科技综合防范体系一期工程，构建数字化、网络化、智能化的社会治安综合防范体系，为人民群众营造更加和谐稳定的社会环境。

四、切实加强政府自身建设

新形势、新任务对政府工作提出了新的更高的要求。我们要牢记立党为公、执政为民的宗旨，牢固树立“一心一意为民、一丝不苟干事、一尘不染从政”的执政理念，不断加强政府自身建设，提高推动科学发展、服务人民的能力和水平，开创政府各项工作新局面。

(一)坚持解放思想，努力做到开拓创新。适应国际国内宏观形势变化，顺应人民群众新期待，坚持以科学发展观为指导，解放思想、创新思维、与时俱进。进一步强化机遇意识、忧患意识、责任意识、创新意识，以食不甘味、寝不能安的责任感，以义不容辞、责无旁贷的使命感，以只争朝夕、奋发有为的精神，集中精力抓发展，一丝不苟干事业。坚持以科学的理念、创新的方法推动工作落实，以勇于改革、锐意进取的气魄破解前进道路上的各种问题和挑战，在加快资源型城市转型、推进科学发展示范区建设上不断探索新路子、实现新突破。适应科学发展示范区建设要求，加强公务员队伍教育培训，努力建设学习型政府。

(二)坚持依法行政，不断提高政府公信力。认真落实《中共唐山市委科学执政、民主执政、依法执政实施纲要》。严格执行人大及其常委会的决议决定，自觉接受人大及其常委会的法律监督、工作监督和政协的民主监督，认真办理人大代表、政协委员的批评、意见、建议和提案，诚恳听取各民主党派、工商联和无党派人士的意见和建议，自觉接受社会各界和群众监督，确保行政权力依法公开透明运行。进一步健全完善社情民意反映制度、重大行政行为合法性事前审查制度和重大事项专家咨询、风险评估制度，社会公示、听证和质询制度，促进科学决策、民主决策、依法决策。深入推进行政执法责任制，落实执法过错责任追究和执法行为评议考核制度，严格按照法定权限和程序行使权力、履行职责。深入开展法制宣传教育，提高公职人员的法制观念和法律素质。

(三)坚持转变职能，推进政府工作提质增效。以政府机构改革为契机，进一步理顺和强化部门职责，提速工作过程，提高工作效率，加快职能转变、提升服务水平。在加强经济调节、市场监管的同时，更加注重社会管理和公共服务，更加注重强化执行和执法职责，切实做到不缺位、不错位、不越位，努力实现优质高效。巩固扩大“干部作风建设年”活动成果，大力发扬求真务实精神，继续发扬“白加黑、五加二和重点项目建设三班倒”的工作作风，少说多做，埋头苦干，多做打基础、管长远的工作。健全政府机关绩效考核体系，完善目标管理责任制。全面践行“一线工作法”，从政府各部门做起深入基层办实事、解难题、搞服务。进一步精简会议、文件，切实改进文风、会风，严格控制各种检查、评比、达标活动，集中更多的精力抓落实、抓发展。

(四)坚持以人为本，尽心竭力为民谋福祉。强化“人民至上、群众第一”的理念，始终把关注民生、改善民生作为政府工作的出发点和落脚点，一心一意想着群众、一心一意依靠群众、一心一意为了群众，真正把工作着力点更多地放在解决群众最关心的热点难点问题上，把工作的关注点更多地放在维护广大人民群众利益上，把公共财力更多地投入到改善民本民生上。坚持深入基层、深入群众调查研究，时刻了解群众的所思、所想、所盼，凝聚民智、加快民富、改善民生，以实实在在的政绩取信于民、造福于民。

(五)坚持从严治政，坚决预防和惩治腐败。深入推进政府系统惩治和预防腐败体系建设，认真落实党风廉政建设责任制，健全和落实“一岗双责”制度。按照市委“六个大力倡导、六个坚决反对”的要求，坚持不懈开展党风廉政教育，严格遵守廉洁自律各项规定和要求，教育引导政府系统广大干部堂堂正正做人，干干净净做事，勤勤恳恳为民。进一步健全完善行政权力运行监控机制，切实加强对重点项目建设、国有资产经营管理、建筑工程招投标、行政审批和直接关系人民群众切身利益、人民群众高度关注的领域和行业的监督管理，从源头上预防和减少腐败。坚持艰苦奋斗、勤俭节约，坚决反对铺张浪费和奢靡之风，努力塑造政府良好形象。

各位代表，宏图凝众志，同心谱华章。让我们更加紧密地团结在以胡锦涛同志为总书记的党中央周围，在省委、省政府和市委的正确领导下，坚定必胜信心，勇敢面对挑战，锐意进取，扎实工作，创造无愧于时代、无愧于历史、无愧于人民的新业绩，为加快建设科学发展示范区和人民群众幸福之都做出新的更大贡献!
